# Comparative Efficacy of Plant Extracts and Probiotics on Growth and Gut Health in Chickens with Necrotic Enteritis

**DOI:** 10.3390/ani14223312

**Published:** 2024-11-18

**Authors:** Ruiting Zhang, Jia Yang, Qingjie Wang, Dandan Hu, Qiping Zhao, Shunhai Zhu, Yu Qiao, Fanghe Zhao, Zhongchuang Wang, Jinwen Wang, Yu Yu, Hongyu Han, Lili Hao, Hui Dong

**Affiliations:** 1 Key Laboratory of Animal Parasitology of Ministry of Agriculture, Shanghai Veterinary Research Institute, Chinese Academy of Agricultural Sciences, Minhang, Shanghai 200241, China; 2218302044@st.gxu.edu.cn (R.Z.); dashiliuzi@163.com (J.Y.); zqp@shvri.ac.cn (Q.Z.); zhushunhai@shvri.ac.cn (S.Z.); qiaoyu540447420@163.com (Y.Q.); xzfh824@163.com (F.Z.); zxclkjhf1999601@163.com (Z.W.); 82101221311@caas.cn (J.W.); yy21058@163.com (Y.Y.); hhysh@shvri.ac.cn (H.H.); 2 College of Animal Science and Technology, Guangxi University, Nanning 530004, China; hudandan@gxu.edu.cn; 3 College of Animal Husbandry and Veterinary Medicine, Southwest Minzu University, Chengdu 610041, China; 4 Shaanxi Provincial Center for Animal Disease Prevention and Control, Xi’an 710003, China; wqj21006@163.com

**Keywords:** plant extracts, necrotic enteritis, antibiotic alternatives, chicken, growth performance, intestinal health

## Abstract

Necrotic enteritis (NE), caused by *Clostridium perfringens*, represents a major economic challenge to poultry production. This study evaluated the effects of four plant extracts—*Astragalus*, pomegranate peel, *Sophora flavescens*, and *Artemisia annua*—in combination with *B. subtilis*, as potential antibiotic alternatives for controlling NE. Using an experimentally induced NE model produced by coinfection with *Eimeria maxima* and *C. perfringens*, 288 chickens were divided into 12 groups to assess growth performance, intestinal lesions, and inflammatory markers. Compared with the enramycin group, the pomegranate peel extract group had a 9.9% increase in body weight gain (BWG), significant enhancement in *claudin-2* expression, and reduced production of inflammatory factors. Compared with the infected control group, the combination of *A. annua* extract and *B. subtilis* resulted in the lowest intestinal lesion scores. The *claudin-2*, *occludin*, and zonula occludens-1 expression levels in the group treated with *S. flavescens* extract combined with *B. subtilis* increased to the highest levels compared to those in the healthy control group. These findings emphasize the potential of plant extracts, particularly pomegranate peel and *A. annua* with *B. subtilis*, as natural alternatives for NE management in poultry, providing a promising approach to reducing reliance on antibiotics.

## Simple Summary

Necrotic enteritis, a severe intestinal disease in chickens caused by bacteria and parasites, poses a major challenge in poultry farming. Traditionally, antibiotics have been used to control this disease, but due to rising antibiotic resistance and regulatory restrictions, alternative treatments are needed. This study explored the use of four plant extracts—*Astragalus*, pomegranate peel, *Sophora flavescens*, and *Artemisia annua*—combined with a beneficial bacterium, *Bacillus subtilis*, to manage necrotic enteritis in chickens. Researchers found that pomegranate peel was effective, even surpassing a standard antibiotic in reducing inflammation and promoting growth. Some combinations, like *S. flavescens* extract with *B. subtilis*, showed increased tight junction proteins’ expression, while others, like pomegranate peel extract with *B. subtilis*, had reduced growth performance. This research highlights the potential of natural treatments as safe, effective options for farmers to improve chicken health, reduce reliance on antibiotics, and support sustainable poultry production.

## 1. Introduction

Necrotic enteritis (NE) is caused by *Clostridium perfringens* and is one of the most important and economically devastating infectious diseases in the global chicken industry [1,2]. The industry incurs annual costs exceeding USD 6 billion due to control measures, treatment, and lost production [3]. The *Eimeria* species belong to the phylum Apicomplexa, including *Eimeria tenella*, *Eimeria acervulina*, *Eimeria maxima*, *Eimeria brunetti*, *Eimeria mitis*, *Eimeria necatrix*, and *Eimeria praecox*, and parasitize in the intestines of chickens [4]. The consequences of infection include malabsorption, enteritis, and, in severe cases for some *Eimeria* species, mortality [5]. Among these species, *E. maxima* infection is considered one of the main factors inducing necrotic enteritis [6]. *Eimeria*-induced NE is associated with epithelial damage, which releases nutrients and serum, or triggers mucogenesis, creating a favorable environment for the colonization and growth of *C. perfringens* [7]. Outbreaks of coccidiosis often occur at the same time as NE [8]. For several decades, antibiotics and other drugs have been used preventively to control NE in commercial poultry [9]. However, due to the global movement to ban antibiotic use in animal food production and the emergence of drug resistance, NE has resurfaced as a serious threat to the poultry industry [10,11]. Consequently, there is an urgent need to identify natural alternatives for managing this disease and enhancing growth and production.

Plant extracts and probiotics are regarded as safe and effective natural alternatives, demonstrating growth-promoting activity, immunomodulation, pathogen inhibition, and antibacterial potential against a wide range of pathogens [9,12]. Plant extracts disrupt cell walls, membranes, proteins, and DNA of bacteria [13,14]. Previous reports showed that *Astragalus* polysaccharides extracted from *Astragalus* roots and artemisinin extracted from the stems and leaves of *Artemisia annua* improved the growth performance of chickens infected with *Eimeria* spp. and proved to be effective against NE [15,16,17,18]. In addition, pomegranate peel, which mainly contains polyphenols and ellagic acid, and *Sophora flavescens*, which contains matrine and oxymatrine, also exert inhibitory effects on *Eimeria* infections [19,20]. *Bacillus subtilis*, a probiotic bacterium, has antibacterial properties and promotes health through active substance production, competitive exclusion, immune enhancement, and digestion improvement [21,22,23,24]. However, the benefits of individual plant extracts or *B. subtilis* are often limited [25]. For example, in a previous report, the combination of *Nigella sativa* and *Kefir* resulted in a mortality rate of 14.06%, whereas the separate administration of *N. sativa* and *Kefir* yielded mortality rates of 31.25% and 23.44%, respectively [26]. Consequently, the synergistic application of a plant extract alongside probiotics may provide a more effective approach for addressing chronic NE.

Currently, there have been no reports on the independent treatment of NE with *S. flavescens* extract and pomegranate peel extract, as well as the effect evaluation of the combination of four plant extracts (*Astragalus*, pomegranate peel, *S. flavescens*, and *A. annua*) with *B. subtilis*. Therefore, in this article, this study utilized a co-infection model involving *E. maxima* and *C. perfringens*, which induced NE, to assess the effects of four plant extracts used alone or in combination with *B. subtilis* on the growth, intestinal lesions, intestinal inflammation, and other relevant factors in chickens.

## 2. Materials and Methods

### 2.1. Ethics Statement

The animal protocols were approved by the Institutional Animal Care and Use Committee of Shanghai Veterinary Research Institute, Chinese Academy of Agricultural Sciences (SHVRI-SZ-20230423-2).

### 2.2. Plant Extracts, Probiotics, Antibiotics

Four plant extracts, *Astragalus* (major active ingredients: 60% astragalus polysaccharides and 20% astragaloside), pomegranate peel (major active ingredients: 40% polyphenol, 20% ellagic acid, and 20% punicalagin), *S. flavescens* (major active ingredients: 35% matrine and 20% oxymatrine), and *A. annua* (major active ingredient: 98% artemisinin), were purchased from Anhui Xuancheng Baicao Pharmaceutical Co. Ltd. (Xuancheng, China). *B. subtilis* was obtained from Shanghai Chuangbo Ecological Engineering Co. Ltd. (Shanghai, China). Enramycin was purchased from Shandong Victory Biological (Jining, China). These plant extracts, *B. subtilis*, or enramycin were thoroughly mixed into the base diet.

### 2.3. Animals, C. perfringens, and E. maxima

One-day-old healthy three-yellow chickens were purchased from the Shanghai Minyou Poultry Farming Professional Cooperative, and were raised under conditions free of coccidia and antibiotics, with feed and water available ad libitum. Chickens were raised at a temperature of 35 °C and 65% humidity from 1 to 7 days. From 7 to 28 days, the temperature should be maintained above 30 °C, with the humidity controlled at 55%. *E. maxima* (Shanghai) and *C. perfringens* type A (CP11) were isolated and preserved by the Shanghai Veterinary Research Institute, Chinese Academy of Agricultural Sciences. *E. maxima* was maintained by inoculating 2–5-week-old coccidia-free chickens.

### 2.4. Experimental Design

A total of 288 1-day-old chickens were divided into 12 experimental groups based on random allocation and similar body weight. Each treatment group comprised three replicates, with eight chickens in each replicate. The experiment included an untreated and uninfected group (NC), untreated and infected group (PC), and 10 treated and infected groups (Table 1). Enramycin was used as a drug control. All chickens received a standardized basic diet (Table S1), and the 10 treatment groups were administered the corresponding drugs mixed into their feed from the beginning until the end of the experiment. The specific dosages of the additives are outlined in Table 1.

Except for the NC group, each chicken was inoculated with 30,000 sporulated oocysts of *E. maxima* at 14 days of age, and the number of sporulated oocysts was calculated using a McMaster counting chamber. On days 18, 19, and 20, each chicken, except for the NC group, was administered 1 mL CP11 at a concentration of 10^8^ CFU/mL orally, which caused NE. The experiment lasted for 28 days, during which growth performance, jejunal lesions, jejunal villus height and crypt depth, and the expression of intestinal-related factors were recorded.

### 2.5. Growth Performance

The body weight (BW) and feed intake of the chickens were recorded on days 14 and 28 to calculate BW gain (BWG), the BWG rate (BWGR), and the feed conversion ratio (FCR) as follows [27].

### 2.6. Jejunum Lesion Score

At 28 days of age, all chickens were euthanized using the cervical dislocation euthanasia method, and their intestines were harvested for lesion scoring. Intestinal lesions were assessed on a scale from 0 to 4, with 0 indicating no visible lesions; 1+ signifying a thin-walled or fragile small intestine; 2+ representing focal necrosis or ulceration; 3+ indicating more extensive necrotic patches; and 4+ corresponding to severe, widespread necrosis [28].

### 2.7. Jejunal Villus Height and Crypt Depth

Jejunal samples were preserved in 4% paraformaldehyde, and then paraffin embedding, sectioning, and Hematoxylin and Eosin staining were all performed by Shanghai Yuxiu Biotechnology Co. Ltd. (Shanghai, China). From each slice, ten well-preserved intestinal villi were randomly selected for observation under an Olympus microscope (Model BX53; Olympus, Tokyo, Japan). The height of the villi and the corresponding crypt depth were measured using Image-Pro Plus software (version 6.0). Villus height was defined as the vertical distance from the tip of the villus to the villus–crypt junction, while crypt depth was the distance from this junction to the crypt base. The ratio of villus height to crypt depth was then calculated for analysis.

### 2.8. Determination of Intestinal-Related Factors Expression Levels

Total RNA was isolated from the jejunum of each group using TRIzol (Invitrogen, Carlsbad, CA, USA) according to the manufacturer’s instructions. Genomic DNA was removed using RNase-free DNase I (40 U/mg; Takara, Tokyo, Japan). cDNAs were synthesized using a transcriptase kit (Invitrogen).

Specific primers for interleukin (IL)-1β, *IL-6*, tumor necrosis factor (TNF)-α, interferon (IFN)-γ, zonula occludens (ZO)-1, *claudin-2*, *occludin*, and the housekeeping gene β-actin were designed according to reference [29,30,31,32,33] and synthesized by Sangon Biotech (Shanghai) Co. Ltd. (Table 2).

QRT-PCR was conducted following the recommended procedure of the SYBR^®^ Premix Ex Taq™ (Perfect Real Time) kit (Takara, Japan). The experiment was carried out in triplicate. Additionally, each group included three technical replicates, ensuring reliable and consistent results.

### 2.9. Statistical Analysis

Statistical analysis was conducted using SPSS 20.0 software (IBM, Armonk, NY, USA). Multivariate analysis of variance (MANOVA) was performed on all normally distributed data, followed by Duncan’s multiple comparison test to evaluate differences among treatments. The images were generated using GraphPad Prism 10 (San Diego, CA, USA). A significance level of *p* < 0.05 was considered statistically significant.

## 3. Results

### 3.1. All Group Growth Performance

The infected-untreated chickens (PC group) showed significantly reduced BWG (*p* < 0.05) and increased FCR (*p* < 0.05) compared with the uninfected control group (NC). The BWG of all treated groups, except for the PPL+BS group, was significantly increased compared to that of the PC group (*p* < 0.05). The BWG of the PPL group was significantly higher than that of the EN group, increasing 9.9% in BWG (*p* < 0.05). The BWG of the AST, AST+BS, and FLA+BS groups was similar to that of the EN group (*p* > 0.05). However, the BWG of the PPL+BS FLA, AA, AA+BS, and BS groups was significantly lower than that of the EN group, with reductions of 12.9%, 7.9%, 9.9%, 7.9%, and 7.9%, respectively (*p* < 0.05) (Table 3).

The FCR of all treated groups was significantly lower than that of the PC group (*p* < 0.05). But the FCR of the PPL group was similar to that of the NC group (*p* > 0.05), and both were significantly lower than that of the EN group. The FCR of the other treatment groups was similar to that of the EN group (*p* > 0.05) (Table 3).

It is worth noting that we used a low pathogenicity model, and no chickens died from NE.

### 3.2. Jejunal Lesion Scores

**Figure 1:**
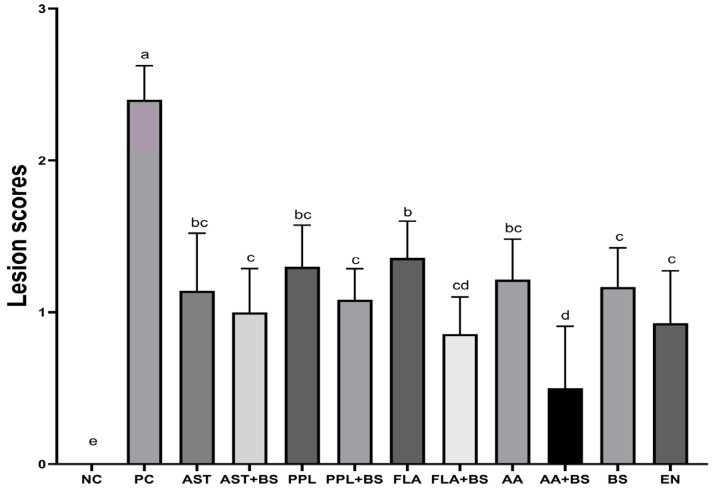
Effects of various supplementation groups on jejunal lesion scores in chickens with necrotic enteritis. a, b, c, d, e, Different letters indicate significant differences (*p* < 0.05). *p* value in Table S2; the specific numerical values for the images can be found in Table S3.

The jejunal lesion scores in the PC group were significantly elevated compared with those in the NC group (*p* < 0.05). All treated groups showed a decrease in lesion scores compared with the PC group (*p* < 0.05). Compared with the EN group, the lesion scores in the FLA group were significantly increased, while that of the AA+BS group was significantly decreased (*p* < 0.05) (Figure 1).

### 3.3. Jejunal Villus Height and Crypt Depth

**Figure 2:**
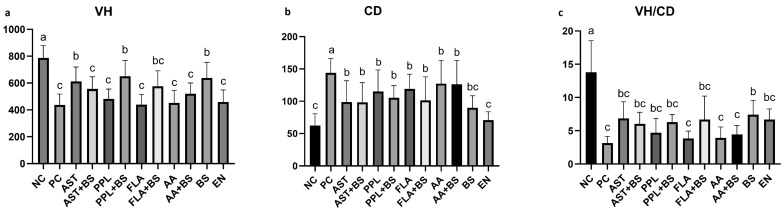
Effects of different supplementation groups on jejunal morphology in 28-day-old chickens. (**a**) VH of intestinal villi. (**b**) Intestinal CD. (**c**) VH/CD ratio in the small intestine. a, b, c, Different letters indicate significant differences (*p* < 0.05). *p* value in Table S2; the specific numerical values for the images can be found in Table S3.

The values for villus height (VH) in all treatment groups were higher than those in the PC group (436 μm), but the AST group (613 μm), PPL+BS group (650 μm), and BS group (637 μm) showed a statistically significant increase compared to the PC group (*p* < 0.05) (Figure 2a). Similarly, the values for crypt depth (CD) in all treated groups and NC group were lower than those in the PC group (114 μm) (*p* < 0.05) (Figure 2b). The villus height/crypt depth (VH/CD) ratio in the PC group (3.1) was significantly lower than that in the NC group (13.8) (*p* < 0.05) (Figure 2c). The VH/CD ratios of all treated groups were higher than that of the PC group, but the differences were not significant (*p* > 0.05) (Figure 2c). The histological staining image of the intestinal villi for each group can be found in Figure S1.

### 3.4. Tight Junction Proteins

**Figure 3:**
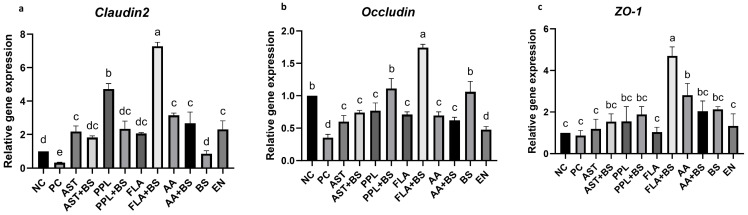
Effects of treatment on expression of tight junction proteins in 28-day-old chickens. (**a**) Expression of *claudin-2* relative to that in the NC group. (**b**) Expression of *occludin* relative to that in the NC group. (**c**) Expression of *ZO-1* relative to that in the NC group. a, b, c, d, e, Different letters indicate significant differences (*p* < 0.05). *p* value in Table S2; the specific numerical values for the images can be found in Table S3.

**Figure 4:**
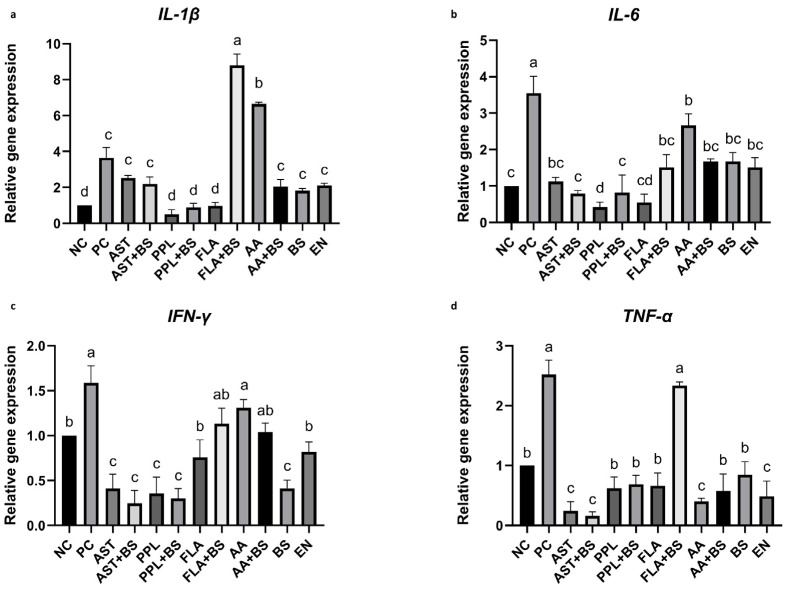
Effects of treatment on expression of proinflammatory cytokines in 28-day-old chickens. (**a**) Expression of *IL-1β* relative to that in the NC group. (**b**) Expression of *IL-6* relative to that in the NC group. (**c**) Expression of *IFN-γ* relative to that in the NC group. (**d**) Expression of *TNF-α* relative to that in the NC group. a, b, c, d, Different letters indicate significant differences (*p* < 0.05). *p* value in Table S2; the specific numerical values for the images can be found in Table S3.

The expression of *claudin-2* and *occludin* in the PC group was significantly decreased compared with that in the NC group (*p* < 0.05), but the expression level of *ZO-1* was similar between the PC group and NC group (*p* > 0.05) (Figure 3a–c). The expression of *claudin-2* in the treated groups was higher than that in the PC group (*p* < 0.05) (Figure 3a). The expression of *claudin-2* in the FLA+BS and PPL groups was significantly higher than that in the EN and other treated groups. However, the expression of *claudin-2* in the BS group was significantly lower than that in the EN group (Figure 4a). The expression of *occludin* in the PPL+BS, FLA+BS, and BS groups was significantly higher than that in the EN group (*p* < 0.05) (Figure 3a). The expression of *claudin-2* in the other treated groups was similar to that in the PC and EN groups (*p* > 0.05) (Figure 3b). The expression of *ZO-1* in the FLA+BS and AA groups was significantly elevated compared with that in the PC and EN groups (*p* < 0.05). The FLA+BS group had the highest expression of *ZO-1*, which was significantly higher than that of all other groups (*p* < 0.05). The expression of *ZO-1* in the other treated groups was similar to that in the PC and EN groups (*p* > 0.05) (Figure 3c).

### 3.5. Proinflammatory Cytokines

The expression of *IL-1β*, *IL-6*, *IFN-γ*, and *TNF-α* in the PC group was significantly increased compared with that in the NC group (*p* < 0.05) (Figure 4a–d). When compared with the PC group, *IL-1β* expression was significantly increased in the FLA+BS and AA groups (*p* < 0.05), significantly decreased in the PPL, PPL+BS, and FLA groups (*p* < 0.05), but not significantly different compared with the other treated groups (*p* > 0.05) (Figure 4a). All treated groups showed significantly lower *IL-6* levels compared with the PC group (*p* < 0.05) (Figure 4b).

*IFN-γ* expression in the FLA+BS, AA, AA+BS, and EN groups was decreased compared with that in the PC group (*p* < 0.05). The *IFN-γ* expression level of the FLA group was similar to that of the NC and EN groups (*p* > 0.05), while the *IFN-γ* expression levels of the other groups are significantly lower than those of the NC and EN groups (*p* < 0.05) (Figure 4c).

*TNF-α* expression in the FLA+BS group was comparable to that in the PC group (*p* > 0.05) (Figure 4d). Its expression in the other treated groups was significantly lower than that in the PC group (*p* < 0.05) (Figure 4d).

## 4. Discussion

Coccidia species predispose towards the exacerbation of NE because *Eimeria* parasites undergo intracellular development in the gut that impairs the intestinal mucosa [34]. Different *Eimeria* species influence NE to different degrees [6]. *E. maxima* is a suitable predisposing factor in experimentally induced NE in chickens, causing physical damage to the upper intestinal epithelium and creating favorable conditions for the colonization and proliferation of *C. perfringens* [35]. So, co-infection with *E. maxima* followed by *C. perfringens* was used to develop our NE model in the present study.

*E. maxima* and *C. perfringens* infections compromise intestinal integrity and trigger inflammation, highlighting the importance of selecting plant extracts with antibacterial and anti-inflammatory properties that also preserve gut integrity. Consequently, *Astragalus*, pomegranate peel, *S. flavescens*, and *A. annua* were selected in this study to evaluate their efficacy against NE by using some clinical indicators, including BWG, FCR, and lesion scores. The intestinal morphology reflects the digestive and absorptive capacity of the intestine [36]. Tight junction proteins are crucial for maintaining the integrity of the intestinal barrier, while proinflammatory cytokines are associated with inflammation, which can affect metabolism and feed consumption [37,38]. So, in this study, these indexes were also measured. We found that the PPL group showed the best growth performance, the AA+BS group had the lowest lesion scores, and the FLA+BS group had the highest tight junction protein expression levels. Although the AST or AST+BS group was effective, their performance was not significantly higher than that of other treatment groups.

*Astragalus* extract contains polysaccharides that possess notable anti-inflammatory, antioxidant, and intestinal epithelial barrier-maintaining properties [39,40]. Previous research has demonstrated that adding *Astragalus* to feed can improve chicken’s BWG and FCR from 1 to 6 weeks, enhance the growth performance of chickens with NE, and modulate levels of tight junction proteins and cytokines [16]. In the present study, the growth performance of chickens fed a diet supplemented with *Astragalus* extract was significantly improved, and the jejunal lesion scores were decreased compared to that of the PC group, which is similar to that of the EN group. In addition, the expression of tight junction proteins *claudin-2* and *occludin* was increased, whereas the expression of cytokines *IL6*, *IFN-γ*, and *TNF-α* was reduced. However, the combination of *Astragalus* extract with *B. subtilis* did not significantly enhance its anti-NE efficacy. The reason may be that both *Astragalus* extract and *B. subtilis* exert their anti-NE effect by regulating the gut microbiota and failing to have a synergistic effect [40].

Pomegranate peel extract is abundant in phenolic compounds and organic acids, which confer substantial antioxidant and antibacterial properties, while lowering intestinal pH to inhibit harmful bacteria [41,42,43]. Previous research found that the addition of 2–4 g of pomegranate peel powder per kilogram feed enhanced BWG and reduced FCR in chickens [44]. In the current study, dietary supplementation with pomegranate peel extract significantly enhanced the expression of *claudin-2* and reduced the expression of proinflammatory cytokines, including *IL-1β*, *IL-6*, *IFN-γ*, and *TNF-α*, outperforming enramycin in its anti-NE effect. However, the coadministration of pomegranate peel extract with *B. subtilis* showed a significant reduction in anti-NE efficacy, suggesting potential antagonistic effects. Previous studies found that a concentration of 0.39 mg/mL of pomegranate peel extract was sufficient to inhibit the growth of *B. subtilis* in vitro [41].

*S. flavescens* extract contains alkaloids, flavonoids, and polysaccharides, providing anti-inflammatory and analgesic benefits, regulating gut microbiota, and repairing intestinal barriers to promote gut health [45,46]. In previous studies, the addition of 200 mg/kg of *S. flavescens* to feed, along with other plant extracts, enhanced chicken growth performance and intestinal barrier function [47]. In this investigation, compared to the PC group, feeding *S. flavescens* extract improved the growth performance of chickens and reduced jejunal lesion scores, the *claudin-2* expression level was upregulated, and *IL-1β*, *IL-6*, *IFN-γ*, and *TNF-α* expression levels were significantly downregulated. But, its anti-NE efficacy was less effective than that of enramycin. The combination of *S. flavescens* extract with *B. subtilis* enhanced BWG and the expression of tight junction proteins, and reduced the lesion scores compared with either agent alone, resulting in the significant improvement of anti-NE efficacy. However, this combination also caused significant elevation in proinflammatory cytokines. The long-term use of *S. flavescens* has been shown to possess hepatotoxicity in mice [48]. Based on the increased inflammatory factors, we speculate that *S. flavescens* extract may exhibit a degree of toxicity in chickens. Moreover, the addition of *B. subtilis* enhances the benefits of *S. flavescens* extract in chickens, but may also accelerate its toxic effects.

*A. annua* extract, primarily composed of artemisinin, possesses antimalarial, antibacterial, and immunomodulating properties [17,18]. Previous studies have indicated that *A. annua* not only possesses efficacy against NE but can also resist coccidial infections [17,49]. In this study, dietary supplementation with *A. annua* extract led to an improvement in BWG and *claudin-2*, *ZO-1*, and *IL-1β* expression levels, and a significant decrease in *IL-6* and *TNF-α* expression levels relative to those in the PC group. However, its anti-NE efficacy was less effective than enramycin. Its combination with *B. subtilis* reduced the jejunal lesion scores, which were lower than that in the enramycin group, highlighting the synergistic potential of this combination in mitigating intestinal damage. *B. subtilis* has been shown to combat intestinal damage [50]. We hypothesize that there is a synergistic interaction between *A. annua* extract and *B. subtilis*.

The previous research showed that plant extracts and probiotics have a synergistic effect. Plant extracts can act as prebiotics to provide substrates for probiotics. In addition, plant extracts metabolized by probiotics are often more easily absorbed and utilized by the host organism [51,52]. In this study, the efficacy of combining four plant extracts with *B. subtilis* for NE treatment was evaluated for the first time. We found a synergistic effect among *S. flavescens* extract and *A. annua* extract with *B. subtilis*. The combination of *Astragalus* extract with *B. subtilis* also leads to a certain enhanced effect in treating NE. However, we found that pomegranate peel extract used in combination with *B. subtilis* exhibited an antagonistic effect. We speculate that pomegranate peel may also have an inhibitory effect on the growth of *B. subtilis* in chicken. Therefore, in the future, we will investigate the effects of pomegranate peel extracts with other probiotics for NE treatment to explore whether there is a synergistic effect.

## 5. Conclusions

The study findings reveal that the independent application of four plant extracts or their co-administration with *B. subtilis* reduced the detrimental effects of NE. Among these plant extracts, the combination of pomegranate peel extract and *A. annua* extract with *B. subtilis* showed the best effect against NE. In addition, it was found that there was a synergistic effect of the combination of *A. annua* extract and *S. flavescens* extract with *B. subtilis*, but an antagonistic effect of the combination of pomegranate peel with *B. subtilis.* These results maybe provide valuable insights into the utilization of feed additives.

## Figures and Tables

**Table 1:** Drug dosage schemes and experimental animal grouping.

Groups	Diet	Groups	Diet
NC	Basal diet	FLA	Basal diet + 0.2 g/kg *Sophora flavescens*
PC	Basal diet	FLA+BS	Basal diet + 0.2 g/kg *Sophora flavescens* + 1 g/kg *B. subtilis*
AST	Basal diet + 0.62 g/kg *Astragalus*	AA	Basal diet + 1 g/kg *Artemisia annua*
AST+BS	Basal diet + 0.62 g/kg *Astragalus* + 1 g/kg *B. subtilis*	AA+BS	Basal diet + 1 g/kg *Artemisia annua* + 1 g/kg *B. subtilis*
PPL	Basal diet + 0.68 g/kg pomegranate peel	BS	Basal diet + 1 g/kg *B. subtilis*
PPL+BS	Basal diet + 0.68 g/kg pomegranate peel + 1 g/kg *B. subtilis*	EN	Basal diet + 10 mg/kg enramycin

**Table 2:** Sequences of primer pairs used for amplification of target and reference genes.

Gene		Primer (5′–3′)	Gene Bank Accession Number
proinflammatory cytokines	*IL-6* [29]	CTGTTCGCCTTTCAGACCTACC	HM179640.1
		GACCACTTCATCGGGATTTATCA	
	*IL-1β* [30]	GGTCAACATCGCCACCTACA	XM_015297469.3
		CATACGAGATGGAAACCAGCAA	
	*TNF-α* [31]	TGTATGTGCAGCAACCCGTA	NM_204267.2
		CCACACGACAGCCAAGTCAA	
	*IFN-γ* [31]	GATGCCACCTTCTCTCACGA	NM_205427.1
		GGATGTCGTGGGTGGTTTTG	
tight junction proteins	*ZO-1* [32]	CTTCAGGTGTTTCTCTTCCTCCTC	XM_413773.4
		CTGTGGTTTCATGGCTGGATC	
	*Occludin* [32]	ACGGCAGCACCTACCTCAA	D21837.1
		GGGCGAAGAAGCAGATGAG	
	*Claudin2* [33]	CTGCTCACCCTCATTGGA	NM_001277622.1
		AACTCACTCTTGGGCTTCTG	
housekeeping gene	*β-Actin* [29]	CCTGGCACCTAGCACAATGAA	NM_205518.2
		GGTTTAGAAGCATTTGCGGTG	

**Table 3:** Effects of different supplementation groups on growth, feed intake, and feed conversion rate in chickens.

Groups	14–28 Days					
	BWG/g	BWGR	*p* Value	FI/g	FCR	*p* Value
NC	1290 ± 60 ^a^	127.7%	1	2360 ± 45	1.830 ^c^	1
PC	810 ± 150 ^e^	80.2%	<0.0001	2260 ± 60	2.795 ^a^	<0.0001
AST	950 ± 120 ^cd^	94.1%	<0.0001	2260 ± 50	2.383 ^b^	<0.0001
AST+BS	950 ± 130 ^cd^	94.1%	<0.0001	2280 ± 45	2.401 ^b^	<0.0001
PPL	1110 ± 120 ^b^	109.9%	<0.0001	2230 ± 70	2.007 ^c^	0.0513
PPL+BS	880 ± 100 ^de^	87.1%	<0.0001	2220 ± 35	2.524 ^b^	<0.0001
FLA	930 ± 100 ^d^	92.1%	<0.0001	2290 ± 60	2.468 ^b^	<0.0001
FLA+BS	1000 ± 160 ^c^	99.0%	<0.0001	2280 ± 50	2.281 ^b^	<0.0001
AA	910 ± 190 ^d^	90.1%	<0.0001	2240 ± 70	2.457 ^b^	<0.0001
AA+BS	930 ± 130 ^d^	92.1%	<0.0001	2230 ± 50	2.403 ^b^	<0.0001
BS	930 ± 160 ^d^	92.1%	<0.0001	2220 ± 80	2.367 ^b^	<0.0001
EN	1010 ± 110 ^c^	1	<0.0001	2390 ± 55	2.371 ^b^	<0.0001

a, b, c, d, e Different letters in the same column indicate significant difference (*p* < 0.05).

## Data Availability

All the data is in the article.

## Supplementary Materials

The following supporting information can be downloaded at: https://www.mdpi.com/article/10.3390/ani14223312/s1, Table S1: Ingredients and composition of the experimental diets. Table S2: The *p*-values for each of the figures. Table S3: The specific numerical values for each figure are as follows. Figure S1: HE-stained slices of the intestine from each group.
