# Supplementary material for: Comparative Efficacy of Plant Extracts and Probiotics on Growth and Gut Health in Chickens with Necrotic Enteritis
Source: Animals (Basel). 2024 Nov 18;14(22):3312. doi: 10.3390/ani14223312 (PMC11591468; doi:10.3390/ani14223312)
Supplement: Supplementary file 1 [file animals-14-03312-s001.zip › TABLES S1 S2 S3.pdf]

Table S1

Ingredients and composition of the experimental diets

Ingredient	Composition (%)
Corn (7.8% CP)	51.38
Soybean meal (46% CP)	40.71
Corn protein flour	0.00
Soybean oil	3.75
Wheat flour	0.00
$CaHPO^3 \cdot 2H^2O$	1.86
Stone powder (37%)	1.24
Sodium chloride	0.35
DL-Methionine (98%)	0.20
L-Lysine HCl (98%)	0.00
Vitamin premix[1]	0.03
Mineral premix[2]	0.20
Choline chloride (50%)	0.25
Sandoquin (Ethoxyquinoline)	0.030
Calculated nutrients[3]	
Metabolizable energy, kcal/kg	2,928.97
Crude protein	21.76
Calcium	1.01
Available phosphorus	0.44
Lysine	1.14
Methionine	0.54

[1]Vitamin premix provided per kg of complete diet: vitamin A (retinylacetate), 9,500 IU; vitamin D_3 (cholecalciferol), 2,500 IU; vitamin E (DL-α-tocopherol acetate), 30 IU; vitamin K_3 (menadione sodium bisulfate), 2.65 mg; vitamin B_{12} (cyanocobalamin), 0.025 mg; biotin, 0.30 mg; folic acid, 1.25 mg; nicotinic acid, 50 mg; D-pantothenic acid, 12 mg; pyridoxine hydrochloride, 6.0 mg; riboflavin, 6.5 mg; thiamine mononitrate, 3.0 mg

[2]Mineral premix provided per kg of complete diet: iron, 80 mg; copper, 8 mg; manganese, 100 mg; zinc, 80 mg; iodine, 0.35 mg; selenium, 0.15 mg

[3]Calculated value based on the analysis of experimental diets

Table S2

The p-values for each of the figures*.

Figure	Group											
	NC	PC	AST	AST+BS	PPL	PPL+BS	FLA	FLA+BS	AA	AA+BS	BS	EN
Figure 1		<0.0001	<0.0001	<0.0001	<0.0001	<0.0001	<0.0001	<0.0001	<0.0001	0.0439	<0.0001	<0.0001
Figure 2a		<0.0001	<0.0001	<0.0001	<0.0001	0.0014	<0.0001	<0.0001	<0.0001	<0.0001	0.0003	<0.0001
Figure 2b		<0.0001	0.0053	0.0064	<0.0001	0.0005	<0.0001	0.0020	<0.0001	<0.0001	0.0815	0.9978
Figure 2c		<0.0001	<0.0001	<0.0001	<0.0001	<0.0001	<0.0001	<0.0001	<0.0001	<0.0001	<0.0001	<0.0001
Figure 3a		0.0468	0.0014	0.0370	<0.0001	0.0003	0.0044	<0.0001	<0.0001	<0.0001	0.9992	0.0004
Figure 3b		<0.0001	<0.0001	0.0094	0.0247	0.5824	0.0033	<0.0001	0.0020	0.0001	0.9727	<0.0001
Figure 3c		>0.9999	>0.9999	0.8024	0.7844	0.1876	>0.9999	<0.0001	0.0003	0.0774	0.0520	0.9912
Figure 4a		<0.0001	<0.0001	0.0015	0.3905	0.9999	>0.9999	<0.0001	<0.0001	0.0057	0.0394	0.0030
Figure 4b		<0.0001	0.9987	0.9559	0.1060	0.9777	0.3111	0.1886	<0.0001	0.2988	0.2598	0.1898
Figure 4c		0.0003	0.0003	<0.0001	<0.0001	<0.0001	0.2538	0.0521	0.0435	0.0999	0.0003	0.0764
Figure 4d		<0.0001	0.0003	<0.0001	0.1141	0.2616	0.1856	<0.0001	0.0039	0.0605	0.9091	0.0159

*All values are compared to the NC group.

Table S3

The specific numerical values for each figure are as follows*.

Figure	Group											
	NC	PC	AST	AST+BS	PPL	PPL+BS	FLA	FLA+BS	AA	AA+BS	BS	EN
Figure 1	0	2.400	1.143	1.000	1.300	1.083	1.357	0.857	1.214	0.500	1.167	0.929
Figure 2a	787.5μm	436.1μm	612.8μm	555.1μm	481.6μm	650.5μm	438.4μm	576.3μm	451.3μm	520.4μm	637.2μm	458.3μm
Figure 2b	62.46μm	144.1μm	98.69μm	98.17μm	115μm	105.3μm	119.2μm	101.5μm	127.3μm	126.1μm	89.92μm	70.58μm
Figure 2c	13.78	3.149	6.849	6.04	4.673	6.27	3.848	6.689	3.904	4.454	7.4	6.665
Figure 3a	1.000	0.326	2.180	1.819	4.722	2.343	2.060	7.279	3.148	2.677	0.859	2.309
Figure 3b	1.000	0.353	0.601	0.739	0.769	1.111	0.709	1.742	0.694	0.619	1.060	0.478
Figure 3c	1.000	0.866	1.188	1.541	1.551	1.887	1.033	4.702	2.807	2.031	2.123	1.335
Figure 4a	1.000	3.658	2.520	2.185	0.504	0.882	0.969	8.794	6.647	2.038	1.816	2.106
Figure 4b	1.000	3.549	1.127	0.794	0.417	0.815	0.550	1.515	2.664	1.679	1.674	1.515
Figure 4c	1.000	1.588	0.410	0.247	0.357	0.300	0.756	1.133	1.311	1.040	0.410	0.820
Figure 4d	1.000	2.519	0.243	0.157	0.620	0.687	0.658	2.335	0.398	0.574	0.844	0.486

*The data presented are the mean values.
